# Biomarkers of diabetic kidney disease

**DOI:** 10.1007/s00125-018-4567-5

**Published:** 2018-03-08

**Authors:** Helen M. Colhoun, M. Loredana Marcovecchio

**Affiliations:** 1 MRC Institute of Genetics & Molecular Medicine, The University of Edinburgh, Western General Hospital, Crewe Road, Edinburgh, EH4 2XU UK; 2 Department of Paediatrics, University of Cambridge, Cambridge, UK

**Keywords:** Biomarker, Diabetic kidney disease, Epidemiology, Nephropathy, Review

## Abstract

Diabetic kidney disease (DKD) remains one of the leading causes of reduced lifespan in diabetes. The quest for both prognostic and surrogate endpoint biomarkers for advanced DKD and end-stage renal disease has received major investment and interest in recent years. However, at present no novel biomarkers are in routine use in the clinic or in trials. This review focuses on the current status of prognostic biomarkers. First, we emphasise that albuminuria and eGFR, with other routine clinical data, show at least modest prediction of future renal status if properly used. Indeed, a major limitation of many current biomarker studies is that they do not properly evaluate the marginal increase in prediction on top of these routinely available clinical data. Second, we emphasise that many of the candidate biomarkers for which there are numerous sporadic reports in the literature are tightly correlated with each other. Despite this, few studies have attempted to evaluate a wide range of biomarkers simultaneously to define the most useful among these correlated biomarkers. We also review the potential of high-dimensional panels of lipids, metabolites and proteins to advance the field, and point to some of the analytical and post-analytical challenges of taking initial studies using these and candidate approaches through to actual clinical biomarker use.

**Electronic supplementary material:**

The online version of this article (10.1007/s00125-018-4567-5) contains a slideset of the figures for download, which is available to authorised users.

## Introduction

Diabetic kidney disease (DKD) and its most severe manifestation, end-stage renal disease (ESRD), remains one of the leading causes of reduced lifespan in people with diabetes [1]. Even early stages of DKD confer a substantial increase in the risk of cardiovascular disease (CVD) [1, 2], so the therapeutic goal should be to prevent these earlier stages, not just ESRD. However, there has been an impasse in the development of drugs to reverse DKD, with many Phase 3 clinical trial failures [3]. The current hard endpoints for the licencing of drugs for chronic kidney disease (CKD) or DKD approved by most authorities, including the US Food and Drug Administration, are a doubling of serum creatinine or the onset of ESRD or renal death. Some of the trial failures are due to insufficient power, with low overall rates of progression to these hard endpoints during the typical trial duration of 3–7 years. As a result, there is increasing interest in the development of prognostic or predictive biomarkers to allow for risk stratification into clinical trials, as well as eventually for targeting preventive therapy. There is also interest in the development of biomarkers of drug response that are surrogates for these harder endpoints. Here we review some of the larger studies published in the last 5 years on prognostic or predictive biomarkers for DKD. Our emphasis is on illustrating some key aspects of the approaches being used recently and what further improvements are needed, rather than systematically reviewing every sporadic biomarker report.

## Biomarkers currently in use

It is well established that the best predictor of future ESRD is the current GFR and past GFR trajectory [4]. Thus, GFR is the most common prognostic biomarker being used for predicting ESRD in both clinical practice and in trials. The Chronic Kidney Disease Epidemiology Collaboration (CKD-EPI) and Modification of Diet in Renal Disease (MDRD) equations, both based on serum creatinine, are commonly used to estimate GFR. The difference in accuracy for staging between CKD-EPI and MDRD is slight, with 69% vs 65% overall accuracy for given stages being found in one study [5]. Serum cystatin C-based eGFR has been proposed as advantageous since, unlike creatinine, it is not related to muscle mass. Equations based on cystatin C overestimated directly measured GFR, while equations based on serum creatinine underestimated GFR in a large study [6]. Others have found that creatinine agrees more closely than cystatin C with directly measured GFR [7]. In those with and without diabetes, cystatin C predicts CVD mortality and ESRD better than eGFR does [8, 9]. However, this may be because factors other than renal function that affect ESRD risk, including diabetes, might also affect serum cystatin C levels, rather than because cystatin C-based eGFR is more accurately measuring GFR itself [10].

Albuminuria strongly predicts progression of DKD but it lacks specificity and sensitivity for ESRD and progressive decline in eGFR. In type 2 diabetes a large proportion of those who have renal disease progression are normoalbuminuric [11, 12]. It has been shown that the coexistence of albuminuria makes DKD rather than non-diabetic CKD more likely in people with type 2 diabetes [13]. However, even in type 1 diabetes, where non-diabetic CKD is much less common, albuminuria was reported to have a poor positive predictive value for DKD as only about a third of those with microalbuminuria had progressive renal function decline [14]. Albumin excretion also had low sensitivity, as only about half of those with progressive renal function decline were albuminuric [14]. Clearly, in evaluating the predictive performance of novel biomarkers, investigators should adjust for baseline eGFR and albuminuria. Historical eGFR data are not always routinely available. Nonetheless, it is important where possible to evaluate whether biomarkers improve prediction on top of historical eGFR.

## Clinical predictors of DKD in type 1 and type 2 diabetes

Apart from albuminuria and eGFR, other risk factors routinely captured in clinical records can predict GFR decline. These have been systematically well reviewed elsewhere [15]. In brief, established clinical risk factors include age, diabetes duration, HbA_1c_, systolic BP (SBP), albuminuria, prior eGFR and retinopathy status. However, there have been relatively few attempts to build and validate predictive equations using clinical data that would form the basis for evaluating the *marginal* improvement in prediction with biomarkers [16–18]. Those that have attempted this reported C statistics for ESRD or renal failure death or prediction of incident albuminuria in the range 0.85–0.90 in type 2 diabetes [17, 18]. In the Joslin cohorts with type 1 diabetes, eGFR slope, albumin to creatinine ratio (ACR) and HbA_1c_ had a C statistic (not cross-validated) for ESRD of 0.80 [19–21]. In the FinnDiane cohort the best model had a C statistic of 0.67 for ESRD [22]. In the Steno Diabetes Center cohort, HbA_1c_, albuminuria, haemoglobin, SBP, baseline eGFR, smoking, and low-density lipoprotein/high-density lipoprotein ratio explained 18–25% of the variability in decline [23]. In the EURODIAB cohort predictive models for albuminuria included HbA_1c_, AER, waist-to-hip ratio, BMI and ever smoking with a non-cross-validated C statistic of 0.71 [24].

In summary, most studies have reported at least modest C statistics for models that contain clinical risk factors beyond eGFR, albuminuria status and age for renal outcomes in type 1 and 2 diabetes. However, despite this, very few biomarker studies have evaluated the marginal improvement in prediction beyond such factors. In the SUrrogate markers for Micro- and Macro-vascular hard endpoints for Innovative diabetes Tools (SUMMIT) study, for example, while forward selection of biomarkers on top of a limited set of clinical covariates selected a panel of 14 biomarkers as predictive, increasing the C statistic from 0.71 to 0.89, a more extensive clinical risk factor model already had a C statistic of 0.79 and a panel of only seven biomarkers showed an improvement in prediction beyond this [25].

## Novel biomarker studies

Ideally, we seek predictive or prognostic biomarkers of the hard endpoint demanded by drug regulatory agencies (i.e. doubling of serum creatinine or the onset of ESRD or renal death). In practice, since many cohorts do not have the necessary length of follow-up or numbers of incident hard endpoints, many studies have sought biomarkers of intermediate phenotypes such as incident albuminuria, DKD stage 3 or eGFR slopes above a certain threshold (Table 1).

**Table 1:** Main studies on biomarkers and DKD published between 2012 and 2017

Author, ref.	Sample size and population	Study design	DKD stage	Biomarkers	Main results	Adjustments
Single biomarkers or several biomarkers not as a panel						
Burns et al [102]	*N =* 259 (*n* = 194 T1D, *n* = 65 controls)	Cross-sectional	Normoalbuminuria; varying levels of GFR	Urinary angiotensinogen and ACE2 levels, activity of ACE and ACE2	Urinary angiotensinogen and ACE activity associated with ACR	No adjustments
Velho et al [44]	*N =* 986 T1D	Prospective	Varying levels of albumin excretion and GFR	Plasma copeptin	Upper tertiles of copeptin associated with a higher incidence of ESRD	Baseline sex, age, and duration of diabetes
Carlsson et al [103]	*N =* 607 T2D	Prospective	Varying levels of albumin excretion	Plasma endostatin	Endostatin levels associated with increased risk of GFR decline and mortality	Baseline age, sex, eGFR and ACR
Dieter et al [104]	*N =* 135 T2D	Prospective	Proteinuria	Serum amyloid A	Higher serum amyloid A levels predicted higher risk of death and ESRD	UACR, eGFR, age, sex and ethnicity
Wang et al [105]	*N* = 100 (*n* = 80 with T2D, *n* = 20 healthy controls)	Cross-sectional	Varying levels of eGFR and ACR	Serum and urinary ZAG	Serum and urinary ZAG associated with eGFR and UACR, respectively	No adjustments
Pikkemaat et al [47]	*N =* 161 T2D	Prospective	eGFR >60 ml min^−1^ 1.73 m^−2^	Copeptin	Copeptin predicted development of CKD stage 3, borderline significant on adjustment for baseline eGFR	Age, sex, diabetes duration, antihypertensive treatment, HbA_1c_, BMI, SBP
Garg et al [50]	*N =* 91 T2D (including *n =* 30 with prediabetes)	Cross-sectional	Varying levels of albumin excretion	Urinary NGAL and cystatin C	NGAL and cystatin C were significantly higher in participants with vs those without microalbuminuria	No adjustments
Viswanathan et al [52]	*N* = 78 (*n* = 65 T2D, *n* = 13 controls)	Cross-sectional	Varying degrees of albuminuria	Urinary L-FABP	L-FABP inversely associated with eGFR and positively associated with protein to creatinine ratio	No adjustments
Panduru et al [62]	*N =* 1573 T1D	Prospective + Mendelian randomisation	Varying degrees of albuminuria	Urinary KIM-1	KIM-1 did not predict progression to ESRD independently of AER Mendelian randomisation supported a causal link between KIM-1 and eGFR	HbA_1c_, triacylglycerols, AER
Pavkov et al [31]	*N =* 193 T2D	Prospective	Varying levels of albumin excretion, eGFR: ≥60 ml/min in 89% participants	Serum TNFR1 and TNFR2	Elevated concentrations of TNFR1 or TNFR2 associated with increased risk of ESRD	Age, sex, HbA_1c_, MAP, ACR and GFR
Fufaa et al [106]	*N =* 260 T2D	Prospective	Varying levels of albumin excretion and eGFR	Urinary KIM-1, L-FABP, NAG and NGAL	NGAL and L-FABP independently associated with ESRD and mortality	Baseline age, sex, diabetes duration, hypertension, HbA_1c_, GFR, ACR
Bouvet et al [107]	*N =* 36 T2D	Cross-sectional	Normoalbuminuria and macroalbuminuria	Urinary NAG	Higher NAG levels associated with microalbuminuria	No adjustments
Har et al [40]	*N =* 142 T1D	Cross-sectional	Varying levels of eGFR Normoalbuminuria	Urinary cytokines/chemokines	Increased urinary cytokine/chemokine excretion according to filtration status with highest levels in hyperfiltering individuals, although not significant after adjustments	Glycaemia
Petrica et al [108]	*N =* 91 (*n* = 70 T2D, *n* = 21 controls)	Cross-sectional	Normoalbuminuria and microalbuminuria	Urinary α_1_-microglobulin and KIM-1 (proximal tubule markers), nephrin and VEGF (podocyte markers), AGE, UACR and serum cystatin C	Significant association between biomarkers of proximal tubule dysfunction and podocyte biomarkers (independently of albuminuria and renal function)	UACR, cystatin C, CRP
Wu et al [109]	*N =* 462 T2D	Cross-sectional	Varying levels of albumin excretion	Serum Klotho, NGAL, 8-iso-PGF2α, MCP-1, TNF-α, TGF-β1	Klotho and NGAL associated with ACR	No adjustments
Sabbisetti et al [58]	*N =* 124 T1D	Prospective	Proteinuria CKD 1-5	Serum KIM-1	KIM-1 associated with eGFR slopes and progression to ESRD	Baseline ACR, eGFR, and HbA_1c_
Velho et al [45]	*N =* 3101 T2D	Prospective	Albuminuria	Plasma copeptin	Copeptin independently associated with renal events (doubling of creatinine or ESRD)	Baseline sex, age, diabetes duration, hypertension, diuretics use, HbA_1c_, eGFR, triacylglycerols, HDL-cholesterol, AER
do Nascimento et al [110]	*N =* 101 (*n =* 19 prediabetes, *n* = 67 diabetes [T1D, T2D] and *n* = 15 controls)	Cross-sectional	Varying levels of albumin excretion	Urinary mRNA levels of podocyte-associated proteins (nephrin, podocin, podocalyxin, synaptopodin, TRPC6, α-actinin-4 and TGF-β1)	Urinary nephrin discriminated between the different stages of DKD and predicted increases in albuminuria	No adjustments
Boertien et al [46]	*N =* 1328 T2D	Prospective	Varying degrees of albuminuria and eGFR	Copeptin	Copeptin associated with change in eGFR independently of baseline eGFR. This association not present in those on RASi	Age, sex, diabetes duration, antihypertensive use, HbA_1c_, cholesterol, BP,BMI, smoking
Lopes-Virella et al [33]	*N =* 1237 T1D	Prospective	Normoalbuminuria	Serum E-selectin, IL-6, PAI-1, sTNFR1, TNFR2	TNFR1 and TNFR2 and E-selectin best predictors of progression to macroalbuminuria	Treatment allocation, baseline AER, ACEi/ARB use, retinopathy cohort, sex, age, HbA_1c_, diabetes duration
Panduru et al [111]	*N* = 2454 (*n* = 2246 T1D, *n* = 208 controls)	Prospective	Varying degrees of albuminuria	Urinary L-FABP	L-FABP was an independent predictor of progression at all stages of DKD, but L-FABP did not significantly improve risk prediction above AER	Baseline WHR, HbA_1c_, triacylglycerols, ACR
Araki et al [53]	*N =* 618 T2D	Prospective	Varying levels of albumin excretion, serum creatinine ≤ 8.8×10^−2^ mmol/l	Urinary L-FABP	L-FABP associated with decline in eGFR	Age, sex, BMI, HbA_1c_, cholesterol, triacylglycerols, HDL-cholesterol, hypertension, RASi use, BP
Lee et al [112]	*N =* 380 T2D	Prospective	Varying levels of albumin excretion	Plasma TNFR1 and FGF-23	FGF-23 was associated with increased risk of ESRD, only in unadjusted model	Sex, baseline diabetes duration, HbA_1c_, eGFR, AER
Cherney et al [41]	*N =* 150 T1D	Cross-sectional	Normoalbuminuria	42 urinary cytokines/chemokines	IL-6, IL-8, PDGF-AA and RANTES levels differed across ACR tertiles	No adjustments
Conway et al [60]	*N =* 978 T2D	Prospective	Varying degrees of albuminuria and eGFR	Urinary KIM-1 and GPNMB	KIM-1 and GPNMB associated with faster eGFR decline, only in unadjusted models Higher KIM-1 associated with mortality risk, only in unadjusted models	Baseline eGFR, ACR, sex, diabetes duration, HbA_1c_, BP
Nielsen et al [48]	*N =* 177 T2D	Prospective	Proteinuria	Urinary NGAL and KIM1 and plasma FGF23	Higher levels of the biomarkers associated with a faster decline in eGFR, although this was not independent of known promoters	Age, sex, HbA_1c,_ SBP and urinary albumin
Jim et al [113]	*N* = 76 (*n* = 66 T2D, *n* = 10 controls)	Cross-sectional	Normoalbuminuria and microalbuminuria	Urinary nephrin levels	Nephrinuria occurred before the onset of microalbuminuria	No adjustments
Gohda et al [30]	*N =* 628 T1D	Prospective	Normal renal function; normoalbuminuria and microalbuminuria	TNFR1 and TNFR2	TNFR1 and TNFR2 strongly associated with risk for early renal decline	HbA_1c_, AER, and eGFR
Niewczas et al [29]	*N =* 410 T2D	Prospective	CKD 1-3	Plasma TNF-*α*, TNFR1, and TNFR2, ICAM-1, VCAM-1, PAI-1, IL-6 and CRP	TNFR1 and TNFR2 were strongly associated with risk of ESRD	Age, HbA_1c_, AER, and eGFR
Fu et al [49]	*N* = 112 (*n* = 88 with T2D, *n* = 24 controls)	Cross-sectional	Varying degrees of albuminuria	Urinary KIM-1, NAG, NGAL	Higher levels of the three markers in T2D than controls. Positive association of NGAL and NAG with ACR; negative association of NGAL and eGFR	No adjustments
Nielsen et al [59]	*N =* 63 T1D	Prospective	Varying levels of albumin excretion and GFR	Urinary NGAL, KIM-1 and L-FABP	Elevated NGAL and KIM-1 were associated with faster decline in GFR, but not after adjustments for known progression promoters	Age, sex, diabetes duration, BP, HbA_1c_, AER
Kamijo-Ikemori et al [51]	*N =* 552 (*n* = 140 T2D and *n* = 412 controls)	Cross-sectional and prospective	Varying degrees of albuminuria and GFR	Urinary L-FABP	L-FABP associated with progression of nephropathy	Age, sex, HbA_1c_, albuminuria status at baseline, BP
Vaidya et al [61]	*N =* 697 (*n* = 659 T1D, *n* = 38 controls)	Cross-sectional and prospective	Varying levels of albumin excretion	Urinary IL-6, CXCL10/IP-10, NAG and KIM-1	KIM-1 and NAG both individually and collectively were significantly associated with regression of microalbuminuria	Age, sex, AER, HbA_1c_, SBP, renoprotective treatment and cholesterol
Panel of biomarkers /proteomics signatures						
Coca et al [114]	*N* = 1536 (*n* = 1346 T2D, *n* = 190 controls)	Nested case–control study and prospective	CKD at various stages	TNFR1, TNFR2 and KIM-1	Higher levels of the three biomarkers associated with higher risk of eGFR decline in persons with early or advanced DKD	Clinical variables
Bjornstad et al [69]	*N =* 527 T1D	Prospective	Varying levels of albumin excretion and eGFR	Plasma biomarkers	B2M, cystatin C, NGAL and osteopontin predicted impaired eGFR	Age, sex, HbA_1c_, SBP, LDL-cholesterol, baseline log ACR and eGFR
Peters et al [70]	*N =* 354 T2D	Prospective	Varying levels of albumin excretion and eGFR	Plasma ApoA4, ApoC-III, CD5L, C1QB, complement factor H-related protein 2, IGFBP3	ApoA4, CD5L, C1QB and IBP3 improved the prediction of rapid decline in renal function independently of recognised clinical risk factors	Age, diabetes duration, diuretic use, HDL-cholesterol
Mayer et al [66]	*N =* 1765 T2D	Prospective	CKD at various stages	YKL-40, GH-1, HGF, matrix metalloproteinases: MMP2, MMP7, MMP8, MMP13, tyrosine kinase and TNFR1	Biomarkers explained variability of annual eGFR loss by 15% and 34% (adj *R*^2^) in patients with eGFR ≥60 and <60 ml min^−1^ 1.73 m^−2^ respectively. A combination of molecular and clinical predictors increased the adjusted *R*^2^ to 35% and 64% in these two groups, respectively.	Sex, age, smoking, baseline eGFR, ACR, BMI, total cholesterol, BP and HbA_1c_
Saulnier et al [115]	*N =* 1135 T2D	Prospective	Varying levels of albumin excretion and eGFR	Serum TNFR1, MR-proADM and NT-proBNP	TNFR1, MR-proADM and NT-proBNP improved risk prediction for renal function decline	Age, sex, diabetes duration, HbA_1c_, BP, baseline eGFR and ACR
Looker et al [25]	*N =* 307 (*n* = 154 T2D, *n* = 153 controls)	Nested case–control	CKD 3	207 serum biomarkers	Panel of 14 biomarkers improved clinical prediction (from 0.706 to 0.868)	Age, sex, eGFR, albuminuria, HbA_1c_, ACEi and ARB use, BP, weighted average of past eGFRs, diabetes duration, BMI, prior CVD, insulin use, antihypertensive drugs
Pena et al [116]	*N =* 82 T2D	Prospective	Normoalbuminuria and macroalbuminuria	Plasma peptides	18 peptides (related to PI3K-Akt, VEGF, mTOR, MAPK, and p38 MAPK, Wnt signalling) improved risk prediction for transition from micro to macroalbuminuria (C statistic from 0.73 to 0.80)	Baseline albuminuria status, eGFR, RASi use
Pena et al [64]	*N =* 82 T2D	Prospective	Varying levels of albumin excretion and eGFR	28 biomarkers	MMPs, tyrosine kinase, podocin, CTGF, TNFR1, sclerostin, CCL2, YKL-40, and NT-proCNP improved prediction of eGFR decline when combined with established risk markers	Baseline smoking, sex, SBP, eGFR, use of oral diabetic medication
Foster et al [117]	*N =* 250 T2D	Prospective	Unselected but 54% albuminuric	β-Trace protein and B2M	β-Trace protein associated with ESRD	GFR, albuminuria, age, sex, diabetes duration, hypertension, cholesterol
Agarwal et al [67]	*N* = 87 (*n* = 67 T2D, *n* = 20 controls)	Prospective	CKD 2-4 Varying levels of albumin excretion	17 urinary and 7 plasma biomarkers	Urinary C-terminal FGF-2: strongest association with ESRD Plasma VEGF associated with the composite outcome of death and ESRD	Baseline albuminuria and eGFR
Siwy et al [75]	*N =* 165 T2D	Prospective	Wide ranges of eGFR and urinary albumin	Urinary CDK273	Validation of this urinary proteome-based classifier in a multicentre prospective setting	Albuminuria
Verhave et al [68]	*N =* 83 T1D and T2D	Prospective	Overt diabetic nephropathy	Urinary IL-1β, IL-6, IL-8, MCP-1, TNF-α, TGF-β1, and PAI-1	MCP-1 and TGF-β1 were independent and additive to proteinuria in predicting the rate of renal function decline	Albuminuria
Bhensdadia et al [84]	*N =* 204 T2D	Prospective	eGFR stage 1-2 and normo-/macroalbuminuria	Urine peptides	Haptoglobin to creatinine ratio: best predictor of early renal function decline	Albuminuria, ACEi use
Merchant et al [82]	*N =* 33 T1D	Prospective	Microalbuminuria	Small (<3 kDa) plasma peptides	Plasma kininogen and kininogen fragments associated with renal function decline	No adjustments but stratum matched for eGFR and albuminuria
Roscioni et al [78]	*N =* 88 T2D	Prospective	Normoalbuminuria and microalbuminuria	CKD273 (urine)	Able to detect progression from normo- to micro- and micro- to macroalbuminuria	Baseline albuminuria status, eGFR, RASi use
Zürbig et al [76]	*N =* 35 T1D and T2D	Prospective	Normoalbuminuria; normal eGFR	Urinary CKD273	Early detection of progression to macroalbuminuria: AUC 0.93 vs 0.67 for urinary albumin	Albuminuria
Titan et al [118]	*N =* 56 T2D	Prospective	Macroalbuminuria	Urinary RBP and serum and urinary cytokines (TGF-β, MCP-1 and VEGF)	Urinary RBP and MCP-1: independently related to the risk of CKD progression	Creatinine clearance, proteinuria, BP
Schlatzer et al [83]	*N =* 465 T1D	Nested case–control	CKD 1 Normoalbuminuria	Panel of 252 urine peptides	A panel including Tamm–Horsfall protein, progranulin, clusterin, and α-1 acid glycoprotein improved the AUC from 0.841 (clinical variables) to 0.889	Age, diabetes duration, HbA_1c_, BMI, WHR, smoking, total and HDL-cholesterol, SBP, ACR, uric acid, cystatin C, BP/lipid treatment
Metabolomics						
Niewczas et al [119]	*N =* 158 T1D	Prospective	Proteinuria and CKD 3	Global serum metabolomic profiling	7 modified metabolites were associated with renal function decline and time to ESRD	Baseline HbA_1c_, ACR, eGFR, BP, BMI, smoking, uric acid levels, RASi use, other antihypertensive treatment, and statins
Klein et al [120]	*N =* 497 T1D	Prospective	Normoalbuminuria	Multiple plasma ceramide species and individual sphingoid bases and their phosphates	Increased plasma levels of very long chain ceramide species associated with reduced macroalbuminuria risk	Treatment group, baseline retinopathy, sex, HbA_1c_, age, AER, lipid levels, diabetes duration, ACEi/ARB use
Pena et al [121]	*N =* 90 T2D	Case–control and prospective	Normoalbuminuria and macroalbuminuria	Plasma and urinary metabolomics	Urine hexose, glutamine and tyrosine and plasma histidine and butenoylcarnitine associated with progression from micro- to macroalbuminuria	Albuminuria, eGFR, RASi use
Niewczas et al [122]	*N =* 80 T2D	Prospective nested case–control study	CKD 1-3	78 plasma metabolites (uremic solutes) and essential amino acids	Abnormal levels of uremic solutes and essential amino acids associated with progression to ESRD	Albuminuria, eGFR, HbA_1c_
Sharma et al [123]	*N* = 181 (*n* = 114 T2D, *n* = 44 T1D, *n* = 23 control)	Cross-sectional	Different CKD stages	13 urine metabolites of mitochondrial metabolism	Differences in urine metabolome between healthy controls and diabetes mellitus and CKD cohorts	Age, race, sex, MAP,BMI, HbA_1c_, diabetes duration
Hirayama et al [124]	*N =* 78 T2D	Cross-sectional	Varying levels of albumin excretion	19 serum metabolites	Able to discriminate presence or absence of diabetic nephropathy	No adjustments
Van der Kloet et al [125]	*N =* 52 T1D	Prospective	Normoalbuminuria	Metabolite profiles of 24 h urines	Acylcarnitines, acylglycines and metabolites related to tryptophan metabolism were discriminating metabolites for progression to micro or macroalbuminuria	No adjustments
Ng et al [126]	*N =* 90 T2D	Cross-sectional	Varying levels of eGFR	Octanol, oxalic acid, phosphoric acid, benzamide, creatinine, 3,5-dimethoxymandelic amide and *N*-acetylglutamine	Able to discriminate low vs normal eGFR	Age at diagnosis, age at examination, baseline serum creatinine
Han et al [127]	*N =* 150 (*n* = 120 T2D, *n* = 30 controls)	Cross-sectional	Varying levels of albumin excretion	35 plasma non-esterified and 32 esterified fatty acids	Able to discriminate albuminuria status	No adjustments

8-iso-PGF2α, 8-iso-prostaglandin F2α; ACEi, ACE inhibitors; ACR, albumin-creatinine ratio; Apo, apolipoprotein; ARB, angiotensin receptor blockers; B2M; β_2_-microglobulin; C1QB, complement C1q subcomponent subunit B; CD5L, CD5 antigen-like; CCL2, chemokine ligand 2; CKD, chronic kidney disease; CRP, C-reactive protein; CTGF, connective tissue growth factor; CVD, cardiovascular disease; CXCL10, CXC chemokine ligand-10; DKD, diabetic kidney disease; ESRD, end-stage renal disease; FGF, fibroblast growth factor; GPNMB, glycoprotein non-metastatic melanoma protein B; GH, growth hormone; HGF, hepatocyte growth factor; IGFBP3, insulin-like growth factor binding protein 3; ICAM-1, intercellular adhesion molecule-1; IP-10, inducible protein 10; L-FABP, liver-type fatty acid-binding protein; MAP, mean arterial blood pressure; MAPK, mitogen-activated protein kinases; MCP-1, monocyte chemoattractant protein-1; MMP, matrix metalloproteinase; MR-proADM, mid-regional pro-adrenomedullin; mTOR, mechanistic target of rapamycin; NAG, *N*-acetylglucosamine; NGAL, neutrophil gelatinase-associated lipocalin; NT-proBNP, N-terminal pro-B-type natriuretic peptide; NT-proCNP, N-terminal pro-C-type natriuretic peptide; P13K-Akt, phosphatidylinositol-3-kinase and protein kinase B; PAI-1, plasminogen activator inhibitor-1; PDGF-AA, platelet-derived growth factor-AA; RANTES, regulated on activation, normal T cell expressed and secreted; RASi, renin–angiotensin system inhibitor; RBP, retinol binding protein; SBP, systolic BP; sTNFR1, soluble TNF receptor-1; T1D, type 1 diabetes; T2D, type 2 diabetes; TNFR, TNF receptor; TRPC6, transient receptor potential cation channel subfamily member 6; UACR, urine albumin-to-creatinine ratio; VCAM-1, vascular cell adhesion molecule 1; VEGF, vascular endothelial growth factor; YKL-40, chitinase-3-like protein 1; ZAG, zinc α2-glycoprotein

### Studies testing single biomarkers or small sets of biomarkers

**Fig. 1:**
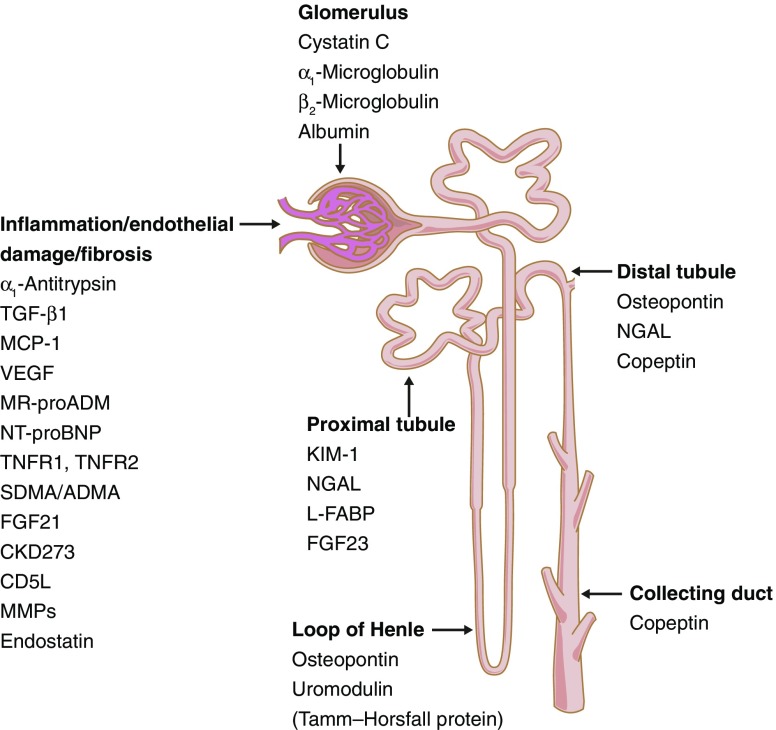
Presumed site of origin of commonly associated biomarkers predictive of DKD. MMPs, matrix metalloproteases. This figure is available as part of a downloadable slideset

Most biomarker reports in the literature are of single candidate biomarkers or small sets of candidate biomarkers that may be assayed in single assays, usually ELISAs, or on multiplexed platforms, such as the Myriad RBM KidneyMAP panel (https://myriadrbm.com/, accessed 17 October 2017). Until recently, most of these studies have taken as their starting point molecules identified from in vitro studies, cell-based studies or animal models. For example, animal models identified kidney injury molecule-1 (KIM-1) [26] and neutrophil gelatinase-associated lipocalin (NGAL) [27]. Candidates studied to date probe pathways thought causal in DKD, such as inflammation, glycation or glycosylation, or endothelial dysfunction. Others focus on glomerular features, such as glycocalyx abnormalities, extracellular matrix deposition, podocyte damage or glomerular fibrosis. Others focus on acute or chronic proximal or distal tubular dysfunction (Fig. 1).

As detailed in Table 1, among these studies of single or few biomarkers, some of the most frequently reported associations with DKD-relevant phenotypes are for biomarkers of inflammation and fibrosis pathways, such as soluble TNF receptors 1 and 2 (sTNFR1 and sTNFR2) [28–33], fibroblast growth factors 21 and 23 (FGF21, FGF23) [25, 34–41] and pigment epithelium-derived factor (PEDF) [42]. Positive associations have also been found for biomarkers of endothelial dysfunction, including mid-regional fragment of proadrenomedullin (MR-proADM) [43], and cardiac injury, including N-terminal pro-B-type natriuretic peptide (NT-proBNP) [43]. Copeptin, a surrogate marker for arginine vasopressin, was associated with albuminuria progression and incident ESRD independently of baseline eGFR in four studies [44–47]. Proximal tubular proteins, such as urinary KIM-1, NGAL [48–50] and liver-type fatty acid-binding protein (L-FABP) [51–53] have been associated with a faster decline in eGFR [48]. The data are most consistent for KIM-1, a protein expressed on the apical membrane of renal proximal tubule cells, with urinary concentrations rising in response to acute renal injury [49, 54–56]. Urinary and blood levels of KIM-1 increased across CKD stages and were associated with eGFR slopes and progression to ESRD during follow-up in some studies [57, 58], but it has not always been a strong independent predictor of progression [59, 60]. There are reports of its association with regression of microalbuminuria in type 1 diabetes [61]. That these associations could reflect a causal role for KIM-1 was suggested by an analysis of the FinnDiane cohort with type 1 diabetes [62]. In this analysis, KIM-1 did not predict progression to ESRD independently of AER. However, using a Mendelian randomisation approach, based on genome-wide association study data for the *KIM-1* gene, an inverse association of increased KIM-1 levels with lower eGFR emerged, suggesting a causal link with renal function.

### Panels of candidate biomarkers

**Fig. 2:**
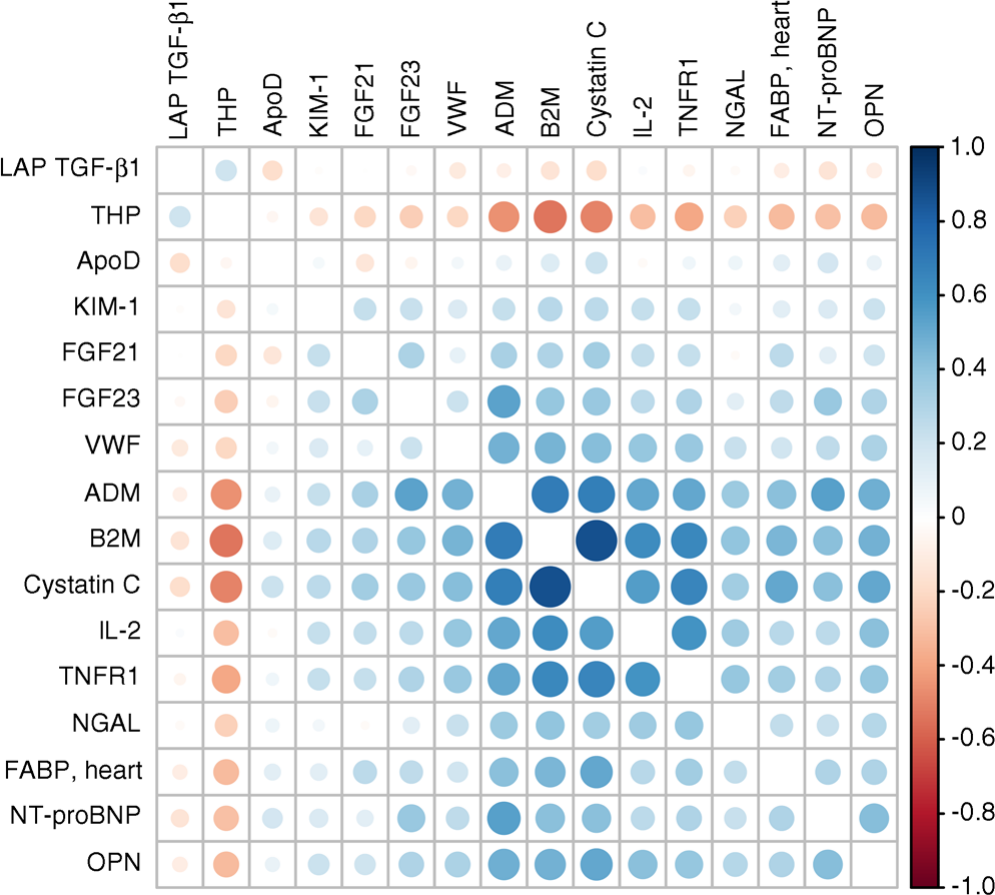
Correlation matrix of biomarker measures in the SUMMIT project (www.imi-summit.eu/) showing there is high correlation between biomarkers that are of interest because of different pathway involvement. ADM, adrenomedullin; FABP, fatty acid-binding protein; LAP TGF-β1, latency-associated-peptide; OPN, osteopontin; THP, Tamm–Horsfall urinary protein; VWF, von Willebrand factor. This figure is available as part of a downloadable slideset

Each of the above biomarkers have some evidence supporting their prediction of renal function decline or other DKD-related phenotypes. However, although they have been investigated as reflecting specific pathways or processes, in reality there are very strong correlations between these biomarkers, even between different pathways. Figure 2 shows the correlation matrix for some of these from the SUMMIT study [25]. Yet, relatively few studies have assayed many of these candidates together to allow the marginal gain in prediction with each additional biomarker to be evaluated. Of those that have, some used a hybrid of discovery and candidate approaches harnessing bioinformatics and systems biology modelling techniques [63]. So, for example, in the SUMMIT study [25], we conducted both data mining and literature review to arrive at sets of candidates that several pathophysiological processes considered relevant for DKD. We assayed these but also a larger set of biomarkers (207 in total) that were already multiplexed with these candidates in the most efficient analysis platforms that were Luminex and mass spectrometry-based. Altogether, 30 biomarkers had highly significant evidence of association with renal function decline when examined singly and adjusted for historical and baseline eGFR, albuminuria and other covariates. In forward selection, 14 biomarkers were selected adjusting for this basic set of covariates (Table 1). On top of a more extensive set of covariates, seven biomarkers were selected: KIM-1, symmetric dimethylarginine/asymmetric dimethylarginine (SDMA/ADMA) ratio, β_2_-microglobulin (B2M), α1-antitrypsin, C16-acylcarnitine, FGF-21 and uracil.

Other such approaches are detailed in Table 1. Of particular note, the Systems biology towards novel chronic kidney disease diagnosis and treatment (SYSKID) consortium used data mining and de novo omics profiling to construct a molecular process model representation of CKD in diabetes [64], choosing ultimately to measure 13 candidates that represented the four largest processes of the model [65]. The panel that gave an increase in prediction of renal disease progression was then reported (C statistic increased from 0.835 to 0.896). In a recent validation study of nine of the biomarkers, the investigators reported that the panel was useful in prediction based on an increase in the adjusted *r*^2^ for the prediction model for eGFR progression from 29% and 56% for those with a baseline eGFR above and below 60 ml min 1.73 m^−2^, respectively, to 35% and 64%, respectively, for the biomarker panel on top of clinical variables [66].

In a study exploring 17 candidate urinary and seven plasma biomarkers in 67 participants with type 2 diabetes, Agarwal et al [67] found that urinary C-terminal FGF-2 showed the strongest association with ESRD, whereas plasma vascular endothelial growth factor (VEGF) was associated with the composite outcome of death and ESRD. The analysis was adjusted for baseline eGFR only and ACR. Of a panel of seven candidates, Verhave et al found that urinary monocyte chemoattractant protein-1 (MCP-1) and TGF-β1 predicted renal function decline independently of albuminuria. Adjustment for baseline eGFR was not made as it surprisingly did not predict decline in univariate testing [68]. In the Coronary Artery Calcification in Type 1 Diabetes (CACTI) study using Kidney Injury Panels 3 and 5, (Meso Scale Diagnostics, www.mesoscale.com/en/products/kidney-injury-panel-3-human-kit-k15189d/ accessed 08 January 2018) containing seven biomarkers, component 2 of a principal component analysis containing B2M, cystatin C, NGAL and osteopontin predicted incident impaired eGFR [69]. Recently, of eight candidate biomarkers studied after adjustment for clinical predictors, apolipoprotein A4 (ApoA4), CD5 antigen-like (CD5L), and complement C1q subcomponent subunit B (C1QB) independently predicted rapid decline in eGFR in 345 people with type 2 diabetes. A notable feature of this study was the adjustment for extensive clinical covariates [70].

Thus, there is some, but not complete, overlap in the explored and selected biomarkers in these panel studies so that further optimisation of a panel of the best reported biomarkers could be considered, especially if it focused on including biomarkers with low correlation with each other. It is also the case that all of the studies, including our own, are too small and there is a need for a large-scale collaboration to increase power, quantify prediction and to demonstrate generalisability [25].

### Discovery ‘omic’ approaches

Apart from candidate biomarkers on multiplexed panels, global discovery or ‘hypothesis-free’ approaches measuring large sets of lipids, metabolites and amino acids, peptides and proteins are increasingly used [71]. The assay methods have most commonly used mass spectrometry-based approaches, but other proteomic methods are now also used [72, 73]. Here we describe some of the main ‘omic’ studies, focusing on whether associations are prospective and whether they have adjusted for baseline eGFR and other relevant covariates.

#### CKD273

This mass spectrometry-based method combines data on 273 urinary peptides into a score that has high accuracy in the cross-sectional classification of eGFR status [74] and has been developed as a commercial test by Mosaique Diagnostics (http://mosaiques-diagnostics.de/mosaiques-diagnostics/, accessed 18 October 2017). Most (74%) of the peptides are collagen fragments, with polymeric-immunoglobulin receptor, uromodulin (Tamm–Horsfall protein), clusterin, CD99 antigen, albumin, B2M, α1-antitrypsin and others comprising the remainder. The collagens, polymeric-immunoglobulin receptor, clusterin, CD99 antigen and uromodulin were lower with worse renal function, whereas the others were higher.

CKD273 was cross-sectionally associated with having albuminuria or/and eGFR <45 ml min^−1^ 1.73 m^−2^ in individuals with type 2 diabetes [75]. In a small study (*n* = 35) of people with type 1 and type 2 diabetes the CKD273 score improved the C statistic for progression to albuminuria to 0.93 compared with 0.67 when using AER, but these data were not fully adjusted for baseline eGFR [76]. In 2672 participants from nine different cohorts, 76.3% with diabetes, CKD273 predicted rapid progression of eGFR better than AER [77]. In a nested case–control analysis, Roscioni et al reported a significant but smaller increase in C statistic for albuminuria incidence that was robust to adjustment for eGFR [78]. The most convincing data to date on the utility of CKD273 come from a subset of 737 samples obtained at baseline in the Diabetic Retinopathy Candesartan Trials (DIRECT)-Protect 2. The CKD273 score was strongly associated with incident microalbuminuria independently of baseline AER, eGFR and other variables. In this study, higher baseline eGFR was associated with incident microalbuminuria, an unusual finding, and CKD273 did not show the expected cross-sectional association with baseline eGFR [79]. Higher CKD273 score at baseline was associated with a larger reduction in ACR in the spironolactone group vs placebo (*p* = 0.026 for interaction) [80]. However, after adjustment for baseline ACR, the interaction between treatment and CKD273 was not statistically significant (*p* = 0.12). The concept that CKD273 will be useful in determining risk of disease progression and may also stratify treatment response to spironolactone is being more definitively tested in the ongoing Proteomic Prediction and Renin Angiotensin Aldosterone System Inhibition Prevention Of Early Diabetic nephRopathy In TYpe 2 Diabetic Patients With Normoalbuminuria (PRIORITY) trial, of 3280 participants with type 2 diabetes [81].

#### Other proteomics

A nested case–control plasma proteomics study yielded kininogen and kininogen fragments as predictors of renal function decline. No adjustment was made for baseline eGFR but stratum matching was used [82]. Using a mass spectrometry approach on 252 urine peptides followed by ELISA validation in a nested case–control design, a panel including Tamm–Horsfall protein (also known as uromodulin), progranulin, clusterin and α-1 acid glycoprotein improved prediction of early decline in eGFR in a cohort of 465 adults with type 1 diabetes, but no adjustment was made for baseline eGFR [83]. In another urinary proteomics study with a very small initial discovery step and then single biomarker validation in 204 participants, haptoglobin emerged to be the best predictor of early renal functional decline but no adjustment for baseline eGFR was made [84].

#### Metabolomics

Several studies have also assessed the potential of metabolomics in the context of DKD. A recent systematic review [85] considered 12 studies (although all included control groups, most were cross-sectional), where a metabolomics-based approach was applied to identify potential biomarkers of DKD. The main metabolites were products of lipid metabolism (such as esterified and non-esterified fatty acids, carnitines, phospholipids), branch-chain amino acid and aromatic amino acid metabolism, carnitine and tryptophan metabolism, nucleotide metabolism (purine, pyrimidine), the tricarboxylic acid cycle or uraemic solutes. The meta-analysis highlighted differences in the results from studies included and this might be related to differences in study population, sample selection, analytical platform.

In the SUMMIT study we used mass spectrometry to measure low-molecular-weight metabolites, peptide and proteins (144 in all) as well as 63 proteins by ELISA and Luminex in a prospective design. Adjusted for extensive covariates, the arginine methylated derivatives of protein turnover ADMA and SDMA, and more strongly their ratio, were independently predictive of rapid progression of eGFR. This ratio, along with metabolites uracil, α1-antitrypsin and C-16 acylcarnitine, were included in the final panel of seven biomarkers [25].

In summary, there are too many global discovery studies in which prediction has not been properly assessed on top of available clinical data, such that replication of findings with proper adjustments is warranted.

#### Genetic biomarkers

Detailed reviews of the literature on genetic biomarkers of DKD have been recently published and are not the focus of this review [86]. In brief, a review of genetic discovery for DKD concluded that “the search for specific variants that confer predisposition to DKD has been relatively unrewarding” [86]. The effect sizes of the reported loci are very small in type 1 [87] and type 2 diabetes [88]. While international meta-analysis of data from the SUMMIT and other consortia are underway, given the effect sizes, it seems very unlikely that genetic risk scores for DKD will contribute usefully as biomarkers for use in the clinical prediction of DKD, even if they may reveal useful insights into pathogenesis.

#### MicroRNAs (miRNAs)

MiRNAs are small non-coding RNA, that block protein translation and can induce messenger RNA degradation, thereby acting as regulators of gene expression [89]. Several studies have assessed urinary and serum miRNA in participants with type 1 and type 2 diabetes in relation to different DKD stages [90–97]. These studies are mostly very small [95] and most have reported simply cross-sectional associations of urinary miRNAs with albuminuria status [91, 93–96]. Three studies have used a nested case–control within prospective cohort design, one of which was in pooled samples [90, 92, 97]. However, there is no overlap in the specific miRNAs being reported as being relevant to DKD. Taken altogether there is not convincing evidence as yet for a clinically useful role for miRNAs in the prediction of DKD progression.

## Are any novel biomarkers actually being used yet?

In reality, despite all the attempts to develop novel prognostic biomarkers, few current trials use biomarkers other than albuminuria or eGFR as stratification variables or entry criteria. An exception is the PRIORITY trial [81], in which the CKD273 panel is being used to risk stratify people into a spironolactone vs placebo arm.

Biomarkers as surrogates of drug response is not the focus of this review but we note that there are also few trials using surrogate biomarkers as endpoints. One ongoing trial is using urinary proteomic panels as a surrogate outcome measure [98]. Another study includes urinary NGAL and KIM-1 as secondary outcome measures [99], and another is using *N*-acyl-β-d-glucosidase, B2M and cystatin C [100]. The SYSKID consortium have argued that past trials have shown that albuminuria/eGFR are insufficient to predict the individual’s response to renoprotective treatments in DKD, and that biomarkers more closely representing molecular mechanisms involved in disease progression and being targeted by therapies are needed [64]. Recently, Pena et al found that urinary metabolites previously shown to be at lower levels in those with DKD than without, decreased in the placebo arm of a trial but remained stable in the arm treated with the endothelin A receptor blocker atrasentan over a short, 12 week trial [101]. Further such studies of changes in biomarkers over time and in response to treatment are needed.

## Future perspectives

In summary, despite the large number of reports in the literature, at present there are few validated biomarkers that have been clearly shown to substantially increase prediction of DKD-related phenotypes beyond known predictors. Few studies have attempted to estimate the marginal improvement in prediction beyond historical eGFR readings that can be expressed as the within-person slope or weighted average past eGFR, as we did in the SUMMIT study [25]. This is an important omission given the increasing availability of electronic healthcare records and potential for applying algorithms to such longitudinal clinical data more easily than measuring biomarkers. Even where some consistency in findings is observed, the extent of publication bias is unknown. Most importantly, biomarkers other than ACR and eGFR are not being routinely used to risk stratify individuals into trials or in clinical practice, despite considerable research investment into DKD biomarkers in recent years.

Large discovery panels have the potential to yield novel biomarkers, but progress has been hampered by small sample sizes, inadequate data analysis approaches (including failure to test the marginal increase beyond established risk factors) and lack of samples for replication. Futhermore, discovery approaches that yield panels of biomarkers measured on different platforms do not lend themselves to an easily implemented single panel in the clinical setting.

If this field is to be advanced, there is a need for a concerted effort to (1) generate and share data on the correlation between existing candidate biomarkers and biomarkers generated from available discovery platforms; (2) generate replication and validation sample and data sets that allow the best panel from available data to be defined; (3) harness the predictive information that exists in clinical records in the era of electronic health record data. Future discoveries should then be evaluated for their marginal prediction on top of clinical data and validated biomarkers.

## Electronic supplementary material

ESM Downloadable slideset (PPTX 333 kb)

## Abbreviations

ACR: Albumin to creatinine ratio
ADMA: Asymmetric dimethylarginine
ApoA4: Apolipoprotein A4
B2M: β_2_-Microglobulin
C1QB: Complement C1q subcomponent subunit B
CD5L: CD5 antigen-like
CKD: Chronic kidney disease
CKD273: CKD classifier based on 273 urinary peptides
CKD-EPI: Chronic Kidney Disease Epidemiology Collaboration
CVD: Cardiovascular disease
DKD: Diabetic kidney disease
ESRD: End-stage renal disease
FGF: Fibroblast growth factor
KIM-1: Kidney injury molecule-1
L-FABP: Liver-type fatty acid-binding protein
MCP-1: Monocyte chemoattractant protein-1
MDRD: Modification of Diet in Renal Disease
miRNA: MicroRNA
MR-proADM: Mid-regional fragment of proadrenomedullin
NGAL: Neutrophil gelatinase-associated lipocalin
NT-proNBP: N-terminal pro-B-type natriuretic peptide
PRIORITY: Proteomic Prediction and Renin Angiotensin Aldosterone System Inhibition Prevention Of Early Diabetic nephRopathy In TYpe 2 Diabetic Patients With Normoalbuminuria
SBP: Systolic BP
SDMA: Symmetric dimethylarginine
SUMMIT: SUrrogate markers for Micro- and Macro-vascular hard endpoints for Innovative diabetes Tools
SYSKID: Systems biology towards novel chronic kidney disease diagnosis and treatment
TNFR: TNF receptor
VEGF: Vascular endothelial growth factor
