# Supplementary material for: Biomarkers of diabetic kidney disease
Source: Diabetologia. 2018 Mar 8;61(5):996–1011. doi: 10.1007/s00125-018-4567-5 (PMC6448994; doi:10.1007/s00125-018-4567-5)
Supplement: Supplementary file 1 — (PPTX 333 kb) [file 125_2018_4567_MOESM1_ESM.pptx]

## Slide 1
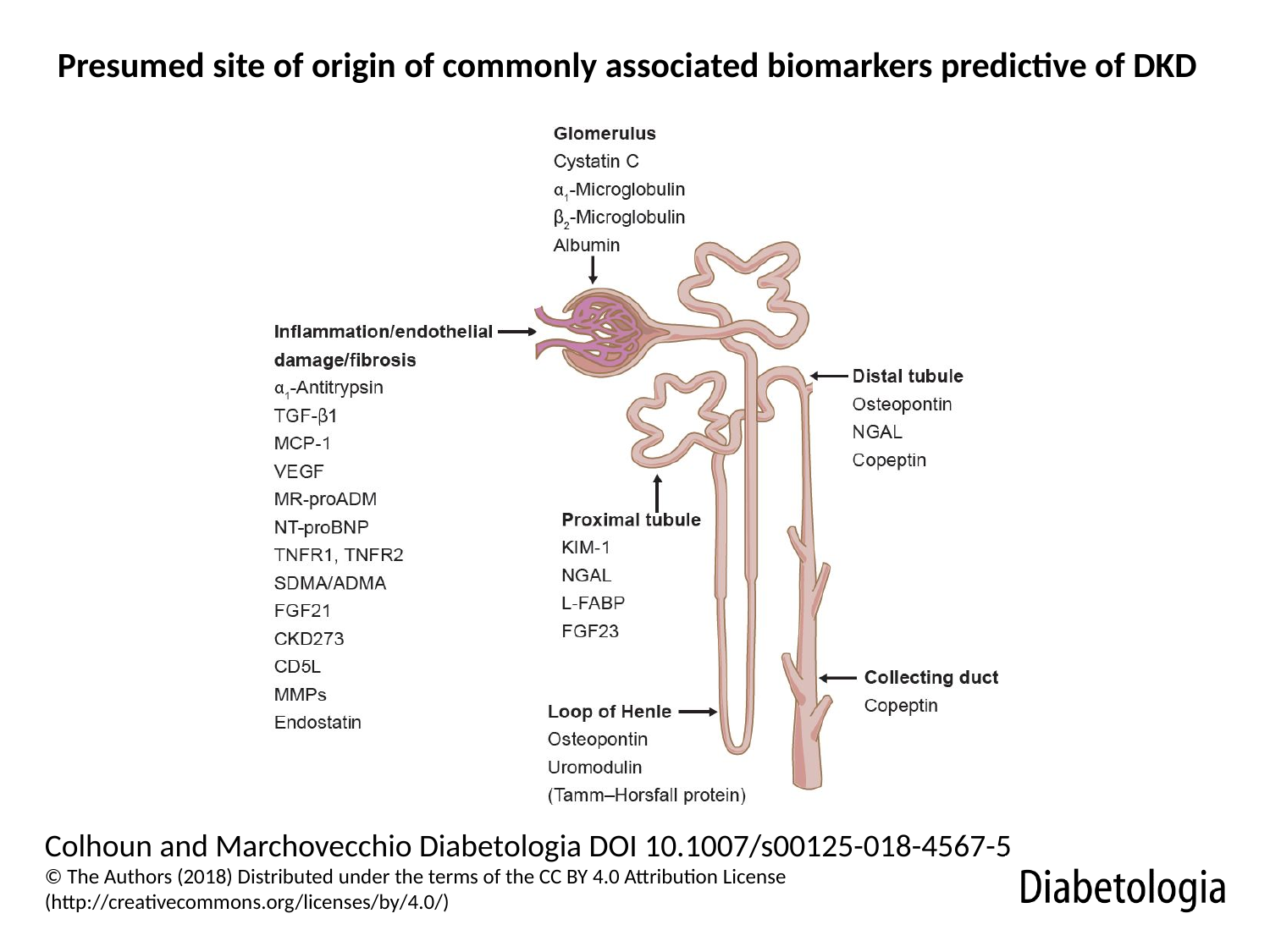

Presumed site of origin of commonly associated biomarkers predictive of DKD
Colhoun and Marchovecchio Diabetologia DOI 10.1007/s00125-018-4567-5
© The Authors (2018) Distributed under the terms of the CC BY 4.0 Attribution License (http://creativecommons.org/licenses/by/4.0/)

## Slide 2
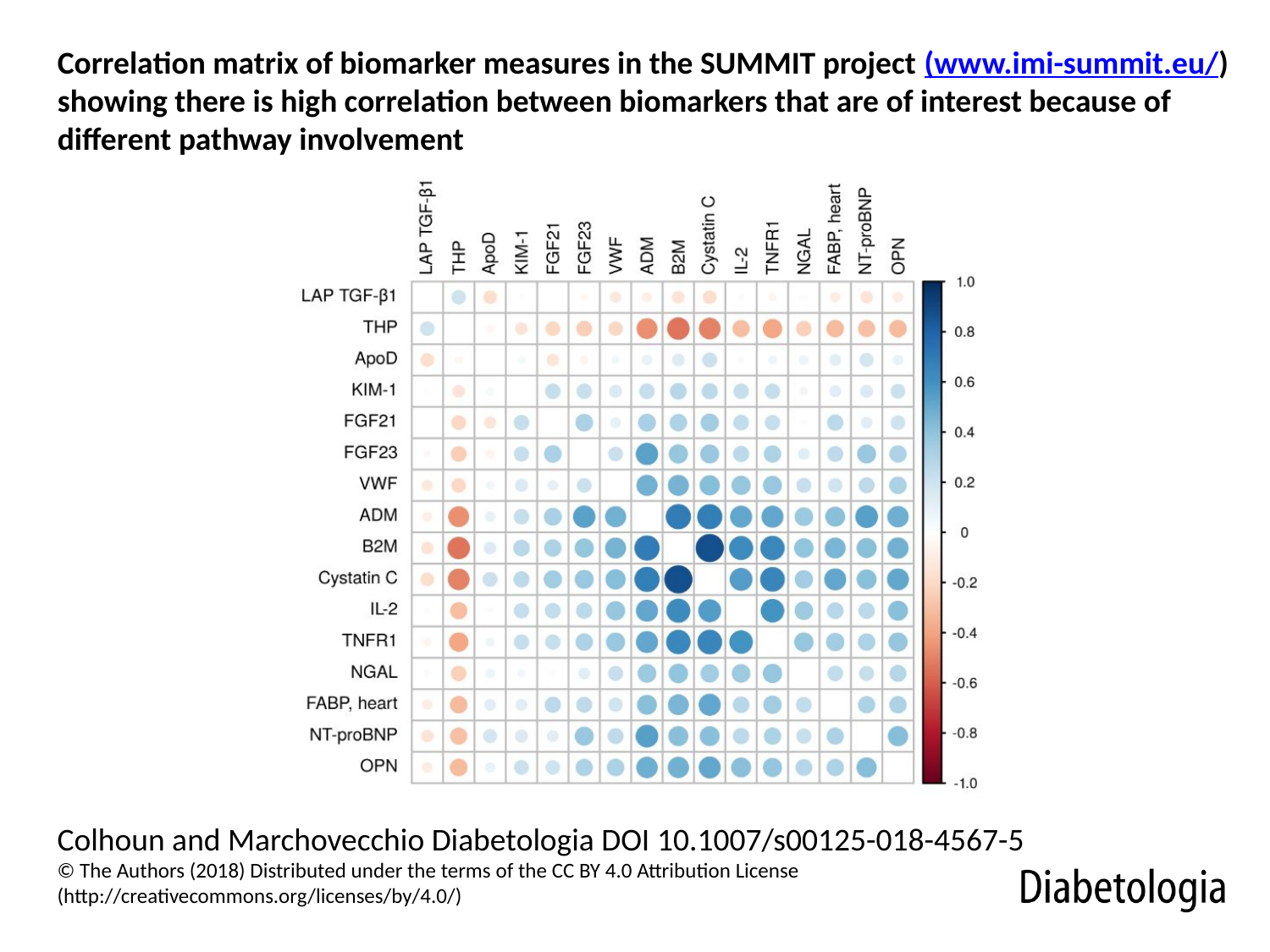

Correlation matrix of biomarker measures in the SUMMIT project (www.imi-summit.eu/) showing there is high correlation between biomarkers that are of interest because of different pathway involvement
Colhoun and Marchovecchio Diabetologia DOI 10.1007/s00125-018-4567-5
© The Authors (2018) Distributed under the terms of the CC BY 4.0 Attribution License (http://creativecommons.org/licenses/by/4.0/)
